# Markedly Elevated Antibody Responses in Wild versus Captive Spotted Hyenas Show that Environmental and Ecological Factors Are Important Modulators of Immunity

**DOI:** 10.1371/journal.pone.0137679

**Published:** 2015-10-07

**Authors:** Andrew S. Flies, Linda S. Mansfield, Chris K. Grant, Mary L. Weldele, Kay E. Holekamp

**Affiliations:** 1 Menzies Research Institute Tasmania, University of Tasmania, Hobart, TAS, Australia; 2 Department of Zoology, Michigan State University, East Lansing, MI, United States of America; 3 Interdisciplinary program in Ecology, Evolutionary Biology and Behavior, Michigan State University, East Lansing, MI, United States of America; 4 Department of Pharmacy and Medical Sciences, University of South Australia, Adelaide, South Australia, Australia; 5 Department of Microbiology and Molecular Genetics, National Food Safety and Toxicology Center, Michigan State University, East Lansing, MI, United States of America; 6 Custom Monoclonals International Corp, West Sacramento, CA, United States of America; 7 Department of Psychology, University of California, Berkeley, CA, United States of America; King's College London, UNITED KINGDOM

## Abstract

Evolutionary processes have shaped the vertebrate immune system over time, but proximal mechanisms control the onset, duration, and intensity of immune responses. Based on testing of the hygiene hypothesis, it is now well known that microbial exposure is important for proper development and regulation of the immune system. However, few studies have examined the differences between wild animals in their natural environments, in which they are typically exposed to a wide array of potential pathogens, and their conspecifics living in captivity. Wild spotted hyenas (*Crocuta crocuta*) are regularly exposed to myriad pathogens, but there is little evidence of disease-induced mortality in wild hyena populations, suggesting that immune defenses are robust in this species. Here we assessed differences in immune defenses between wild spotted hyenas that inhabit their natural savanna environment and captive hyenas that inhabit a captive environment where pathogen control programs are implemented. Importantly, the captive population of spotted hyenas was derived directly from the wild population and has been in captivity for less than four generations. Our results show that wild hyenas have significantly higher serum antibody concentrations, including total IgG and IgM, natural antibodies, and autoantibodies than do captive hyenas; there was no difference in the bacterial killing capacity of sera collected from captive and wild hyenas. The striking differences in serum antibody concentrations observed here suggest that complementing traditional immunology studies, with comparative studies of wild animals in their natural environment may help to uncover links between environment and immune function, and facilitate progress towards answering immunological questions associated with the hygiene hypothesis.

## Introduction

Evolutionary processes have shaped the vertebrate immune system over time, but proximal socio-ecological factors mediate the activation, duration, and intensity of immune responses [1, 2]. For example, an animal’s sex, diet, sociality, life-history stage, and pathogen pressure can influence its immune function [2–7]. One proximal factor widely believed to shape immune function during ontogenetic development is exposure to potential pathogens, including both microbes and macroparasites. In fact, reduced parasitic helminth exposure has been correlated with the increased prevalence of allergy and autoimmune disease in some human populations (reviewed in [8]). The hygiene hypothesis was originally proposed as an explanation for the commonly observed pattern of increased occurrence of allergic disease in relatively hygienic environments [9] and in human populations with access to modern medicine. This hypothesis has been modified many times, but one of its basic tenets remains the idea that reduced exposure to potential pathogens leads to dysregulated development of immune defense pathways, and consequently to increased risk of allergy and autoimmune disease. Although debate about the implications and breadth of the hygiene hypothesis continues, it is clear that environmental exposure to potential pathogens can play a critical role in the development of the immune system [10–12].

Wild-type animals, which are those that putatively represent the normal phenotype for a given species as it occurs in nature, represent a critical component of many laboratory studies, yet few studies have actually examined immune function in wild animals living in their natural environment, and fewer still have compared immune function between wild and captive conspecifics [12–17]. Differences between captive and wild animals with respect to immune activation, duration and intensity can be expected due to regular cleaning of captive facilities, implementation of pathogen control programs, reduced energy expenditure in captive animals, and a more stable environment in captivity than in the wild [6, 18, 19].

A more robust understanding of the effects of ecological variables such as pathogen exposure on immune function might be gained by studying immune function in non-traditional species, and assessing how basic immune defenses differ between wild and captive animals with similar genetic backgrounds. Carnivores are particularly interesting with respect to immune function because they are regularly exposed to pathogens that are capable of infecting both prey and predator. For example, particular *Salmonella spp*. and Shiga toxin-producing *Escherichia coli* are commonly found in wildlife carcasses [20, 21] on which carnivores feed. Furthermore, several carnivores in Africa, including spotted hyenas (*Crocuta crocuta*), regularly test seropositive for anthrax antibodies, yet they show no signs of disease [22]. Wild hyenas are also known to harbor an abundance of parasitic worms of several genera [23], and survive exposure to a wide array of viral pathogens including but not limited to: rabies virus, canine distemper virus, corona virus, calicivirus, and parvovirus [24, 25]. In the wild, spotted hyenas live in large social groups, which increases the probability of acquiring socially transmitted pathogens [26]. Furthermore spotted hyenas can live more than 20 years, which likely makes their acquired immune system, specifically recall responses, more important than in shorter-lived species, such as mice, that are not re-exposed to the same pathogens over the course of many years [27]. High pathogen exposure rates coupled with low mortality rates from infectious disease suggest that immune function may be particularly robust in wild spotted hyenas.

**Fig 1:**
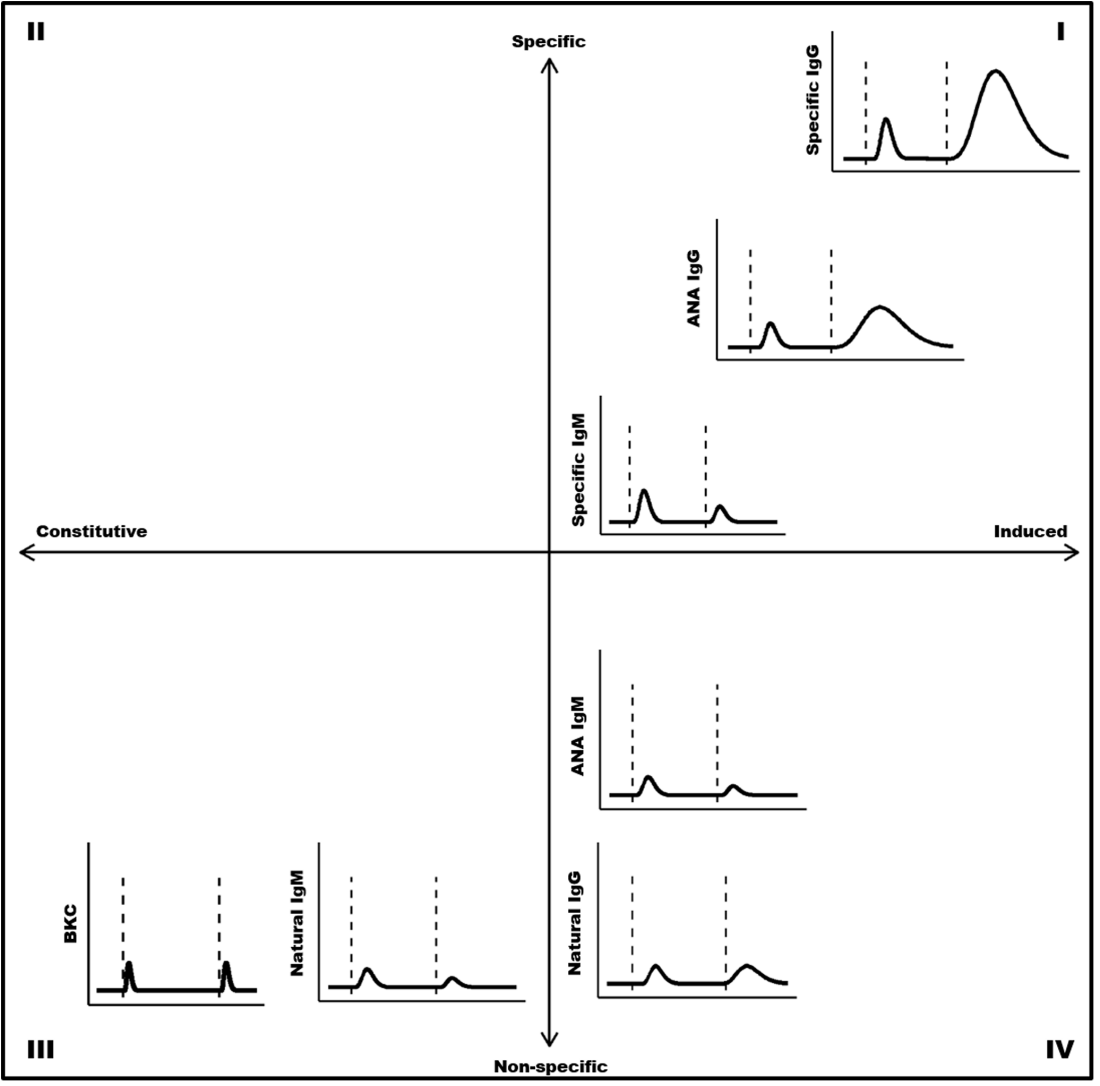
Immune defense component model modified from Schmid-Hempel and Ebert [28]. Patterns shown here are hypothesized based on existing literature and the locations within the plot were adjusted based on the results of this study. Bacterial killing capacity (BKC) is the least specific defense and also falls at the constitutive end of the x-axis. Specific IgG falls near the induced end of the x-axis, and is the most specific defense represented here. Anti-nuclear antibodies (ANAs) are specific to nuclear proteins and nucleic acids and can be induced following ligation of endosomal pattern recognition receptors. Natural IgG and IgM are non-specific and can be produced independent of CD4 T cell help. The dashed lines represent primary exposure (left) and secondary (right) exposure.

Our primary objective in this study was to assess differences in immune function between wild spotted hyenas inhabiting a pathogen-rich and highly variable environment in their natural habitat, and captive hyenas inhabiting a more stable captive environment in which efforts were made to minimize the hyenas’ exposure to pathogens. To accomplish this, we used an immune defense component model (Fig 1), modified from Schmid-Hemp and Ebert [28], to compare immune defenses in wild and captive hyenas along two continua: 1) constitutive to induced defenses, and 2) non-specific to specific defenses. Constitutive defenses are innate defenses that are present in the absence of infection and are functional without prior exposure to a pathogen [28]. Induced defenses can be innate or adaptive and are initiated in response to the recognition of specific molecular patterns that are often associated with threats to physiological homeostasis [28]. Specific defenses are effective against only a limited range of antigens and specificity can be modified during a host’s lifetime. Specific defenses are most effective upon repeated exposure to the same pathogen, because they are maintained after initial infection, allowing a much faster response to subsequent infections. Non-specific defenses target a broader range of antigens but are not modified over time.

Natural antibodies (nAbs) are germline encoded, undergo little or no somatic hypermutation, and are thus largely non-specific, but can be rapidly induced by the microbiota or infection by pathogens [29–32]. By contrast, specific antibodies produced by B-2 cells, are primarily of the IgG isotype in non-mucosal regions, are produced in response to a strong stimulus, such as an infection, and undergo somatic mutation and affinity maturation [31]. nAbs are primarily of the IgM isotype, but can also be of the IgG and IgA isotypes [33, 34]. Natural IgM makes up the majority of total IgM in mice, and functions in concert with complement to provide first line defenses to inhibit pathogen invasion and replication [30]. Anti-nuclear antibodies (ANA) represent a subset of autoantibodies (aAbs) that bind to intracellular self-antigens, and are commonly derived from nAbs independently of help from T cells, but can also be induced via T cell dependent mechanisms [35].

Complement, the primary mediator of serum bacterial killing capacity (BKC), is a first-line immune defense that is continually produced at low levels in the absence of pathogenic challenge, and functions to prevent infection [36, 37]. Complement levels can increase quickly following infection, but the duration of the complement pulse is generally short compared to the rise in antibody levels following infection (Fig 1) [38].

We hypothesized here that higher levels of induced immune defenses (Fig 1: quadrants I and IV) would be observed in a pathogen-rich natural environment than in a relatively clean captive environment. By contrast, we expected that constitutive immune defenses (Fig 1: quadrants II and III), which are less dependent on pathogen exposure and maintained at baseline levels in the absence of such exposure, should be similar in pathogen-rich wild and pathogen-poor captive environments [14, 28]. We measured relative concentrations of total IgG and IgM antibodies, autoreactive IgG (aIgG) and autoreactive IgM (aIgM) that bind nuclear antigens, natural IgG (nIgG) and natural IgM (nIgM) against keyhole limpet hemocyanin (KLH), and complement-mediated bacterial killing capacity (BKC) of serum. Based on these studies we found strikingly different serum antibody concentrations in captive and wild hyenas.

## Materials and Methods

### Collection of serum samples from captive and wild hyenas

Captive spotted hyenas used in this study were born and housed at the Field Station for Behavioral Research at the University of California, Berkeley (UCB) (37.882843°N, -122.240330°E). The colony was founded by importing 20 infant hyenas from the Talek region of Kenya in 1984–1985 [39]. All hyenas were treated with anti-helminthic drugs prior to importation from Kenya and no parasitic worms have been detected in the colony since its founding, whereas a high diversity and abundance of parasitic worms have been detected in the feces of the wild hyenas [23]. The captive hyena enclosures have cement floors that are hosed down each day with water, and steam cleaned annually. Fecal material is removed from medium-sized external enclosures on a daily basis, and fecal material is removed from large outdoor enclosures on a monthly basis. The captive hyenas used in our study have been in captivity for less than four generations, and were derived directly from the same wild population under study here, located in the Talek region of the Masai Mara National Reserve, Kenya.

No disease outbreaks have ever occurred in the captive population, but many virulent viral, bacterial, and protozoan pathogens have been documented in wild spotted hyenas in East Africa [22, 24, 25, 40–42]. Ticks, fleas, tsetse flies, and other biting insects, any of which are capable of transmitting pathogens to hyenas, are commonly observed on wild hyenas, but not on captive hyenas. Additionally, the captive hyenas are fed only USDA approved meat and standard carnivore chow (Nebraska Brand, USA), whereas the wild hyenas feed on fresh ungulate carcasses and rotting carrion [43].

The captive hyenas exhibit most of the same behaviors as wild hyenas, including scent marking, establishment of dominance hierarchies, greeting ceremonies, and mating [39], but social group size is much smaller in the captive group than in the wild group. Pathogen transmission is often positively correlated with host density, and the effect becomes more pronounced in species that live in large aggregations [44]. Wild hyenas live in social groups that can include more than 100 individuals [45]. By contrast, captive hyenas are housed in groups of two or three. The low number of hyenas in each enclosure reduces the possibility of direct transmission of pathogens among group members via social interactions. Interspecific pathogen transmission between captive hyenas and wild local animals is probably rare because wild animals seldom stray into the hyena enclosures. Wild hyenas are regularly observed feeding on carcasses from which lions, jackals, and vultures, have also fed, and wild hyenas often engage in fierce battles with other carnivores.

The Institutional Animal Care and Use Committees at both the University of California, Berkeley and Michigan State University approved this study, under approval numbers # R091-0609R and # 07/08-099-00, respectively. Captive hyenas were immobilized with blow dart-delivered intramuscular injections of ketamine (4–6 mg/kg) and xylazine (1 mg/kg). Blood was drawn from the jugular vein and serum was aliquoted, and frozen at -80°C until use. Sera from wild spotted hyenas in the Maasai Mara, Kenya (-1.44290°N, 35.20731°E), were collected as part of the long-term Michigan State University Hyena Research Project, which has continuously monitored and recorded demographic information about the wild hyena clan since 1988. Research on wild hyenas was approved by the Kenya Wildlife Service and carried out under Research Permit # NCST/RRI/12/1/BS-011/24 from the Kenyan National Council for Science, Technology & Innovation. Hyena tissue samples were exported under the Genetic Materials Access Permit #0004 from the National Environment Management Authority of Kenya. Wild spotted hyenas were immobilized using dart-delivered intramuscular injections of Telazol (6.5 mg/kg; Zoetis, USA) in a plastic dart fired from an air rifle (Telinject Inc., USA) [46]. Serum was collected as described above, but was snap frozen in liquid nitrogen in the field, then transported to Michigan on dry ice, and stored at -80°C until use.

### Quantification of total IgG and IgM

Total IgG and IgM were quantified using sandwich ELISAs. Checkerboard 2-fold dilutions of sera and detection antibodies were used to determine optimal dilutions for assays. First, capture antibodies (IgG: Bethyl Labs # A20-117A; IgM: Custom Monoclonals International # CM7) were diluted into coating buffer (0.05 M carbonate-bicarbonate, pH 9.6) at 10 μg/ml, aliquoted into 96-well plates. The plates were then washed twice with TBST and non-specific binding was then blocked 1% BSA in TBS. All standards, sera, and detection antibodies were diluted in 1% BSA in TBST. We used pooled sera from both captive and wild hyenas to create a standard curve on each plate. Standard curves were run in duplicate on each plate. The starting dilutions of the pooled sera standard curve were 1:10,000 and 1:100 for total IgG and IgM, respectively, and 12 doubling dilutions were performed. Serum samples were diluted 1:100,000 for IgG and 1:1,000 for IgM and 100 μl were aliquoted into each well and incubated for 60 minutes. All samples were tested in triplicate. The plates were washed three times with TBST before adding 100 μl of horseradish peroxidase (HRP)-conjugated anti-IgG (Kirkegaard & Perry Laboratories # 04-20-02) or anti–IgM (Kirkegaard & Perry Laboratories # 04-20-03) detection antibodies to the plates at concentrations of 0.2 μg/ml and 0.25 μg/ml, respectively. The plates were then incubated 60 minutes and washed four times. 100 μl/well of 3,3',5,5'-tetramethylbenzidine (TMB) was used as the substrate for the HRP, and the color change reaction was stopped using 0.2 M H_2_SO_4_. Absorbance was read at 450 nm on a standard plate reader. Relative serum concentrations were calculated using the “calibFit” package in R [47].

### Quantification of natural antibodies against keyhole limpet hemocyanin (KLH)

We used naturally-occurring anti-KLH antibodies as our measure of nAbs [48]. KLH is a standard immunogen in laboratory studies; it is unlikely that either captive or wild hyenas could ever have been exposed to KLH, which is derived from marine gastropods [14]. Natural anti-KLH is detectable in pre-immune human serum, and concentrations are relatively stable over time [49, 50]. nAbs were quantified using the same protocol as above, but with the following changes. Instead of coating capture antibodies to the plates, we coated KLH (EMD Millipore # 374805) at 10 μg/ml. The pooled sera again underwent 12 doubling dilutions, with starting dilutions of 1:50 and 1:12.5 for IgG and IgM, respectively. Serum samples were diluted 1:250 for IgG and 1:100 for IgM, and 100 μl were aliquoted into each well and incubated for 60 minutes. The horseradish peroxidase (HRP)-conjugated anti-IgG or anti–IgM detection antibodies were added to the plates at concentrations of 0.2 μg/ml and 0.25 μg/ml, respectively.

### Quantification of anti-nuclear antibodies (ANA)

We used commercially available *in* vitro diagnostic ANA kits (Hemagen # 6659 96, Columbia, MD, USA) to assess aIgG and aIgM concentrations [51]. Sera were diluted 1:1,600 and 1:400 for IgG and IgM, respectively. We again used HRP-conjugated anti-IgG or anti-IgM detection antibodies added to the plates at concentrations of 0.2 μg/ml and 0.25 μg/ml, respectively. See the manufacturer’s product data sheet for a full description of the test kit.

### Quantification of serum bacterial killing capacity (BKC)

Two strains of bacteria were tested: *Escherichia coli* (ATCC# 8739) was chosen because it has been used as a standard bacterial strain in several other studies of bactericidal capacity [52]; *Proteus mirabilis* (ATCC# 35659) was chosen because, like *E*. *coli*, it is a gram negative bacterium that is commonly found in the gastrointestinal tract of many animals and was more susceptible to complement-mediated killing than most other strains in preliminary testing. All bacterial killing assays were conducted in 96-well round bottom plates. All serum samples from both wild and captive hyenas were passed through a 0.22 μm filter prior to adding to the 96-well plate to remove residual red blood cells and other cellular debris. Sera were then diluted in phosphate buffered saline (PBS), added to row 1 and serially diluted in PBS. 50 μl of bacteria in cation-adjusted Mueller-Hinton broth II (MHBII) was then added to each well [53, 54] to reach a total volume of 100 μl, with the final serum dilutions ranging from 1:8 to 1:1,024 for *E*. *coli* 1:4 to 1:512 for *P*. *mirabilis* Control wells were loaded with bacteria, but no serum, and blank wells were loaded with PBS and MHBII without bacteria.

One day prior to beginning the bacterial killing assay, *E*. *coli* or *P*. *mirabilis* were inoculated into MHBII and incubated overnight at 37°C while shaking at 120 rpm. After overnight incubation, bacterial cell concentration was adjusted to approximately 10^8^ CFU/ml by diluting bacteria in sterile PBS until optical density at 600 nm matched the corresponding turbidity of the McFarland turbidity standard (BD Biosciences # 287298). Bacteria were then serially diluted in PBS to a concentration of 10^4^ CFU/ml. Control wells, serum wells and antibiotic wells were next quickly loaded with 50 μl stock bacteria-containing broth, resulting in a final concentration of 500 CFU/well and a total volume of 100 μl/well. 50 μl of sterile MHB were added to blank wells to bring the total volume of each well to 100 μl. Plates were then placed in an incubator at 37°C and turbidity was measured after 24 hours on a Bio-Tek plate reader at 600 nm. Percent inhibition was calculated by dividing the optical density of each sample well by the mean optical density of the control bacteria wells. Because the assay allows the bacteria to grow to saturation, there was a clear distinction among wells exhibiting bacterial growth and those without growth. The minimum inhibitory concentration (MIC) was defined as the negative log_2_ of the lowest dilution that exhibited over 90 percent inhibition compared to the mean of the control wells [55, 56]. For example, if 1:40 was the lowest dilution exhibiting complete growth inhibition, the response variable value would be:-*log*_*2*_ *(1/40) = 5*.*32*. Additionally, the plates were visually inspected to ensure that there was no bacterial growth at the calculated MIC. The mean of triplicates for each sample was used as the final MIC, and we used the combined mean MIC for both *E*. *coli* and *P*. *mirabilis* as a general measure of serum BKC.

### Statistical analysis

We used version 3.0.3 of the software package R [47] to create linear regression models to assess the effects of captivity on immune function in spotted hyenas. Age and sex are known to affect aspects of immune function in some species, so we included age in months and sex as covariates in all of the models. Because no single model is a perfect representation of nature [57], we used an information theoretic multimodel inference approach rather than choosing a single best model [58]. The full models included captivity status, sex, and age as input variables, as well as all two-way interactions between input variables. We explored all possible subsets of the full model, which yielded 18 candidate models for each dependent variable [58]. All subset models were ranked according to Akaike’s information criteria corrected for small sample size (AICc) [59]. Models with a difference in AICc of less than two (Δ AICc < 2) are considered to be equally good [59]. The top models, those with Δ AICc < 2, were then averaged using the 'MuMIn' package in R to produce weighted averages of regression parameter coefficients, 95% confidence intervals, and p-values for each input variable in the top models [47]. In cases where only a single model had Δ AICc < 2, we report the results directly from the linear model, rather than weighted averages.

The response variables total IgG, total IgM, anti-ANA IgG, anti-ANA IgM, anti-KLH IgG anti-KLH IgM, and BKC were normalized by log transformation; the input variable age was normalized using a square root transformation. All continuous response and predictor variables were then centered and standardized by subtracting the mean and dividing by the standard deviation; standardization allows for more interpretable comparisons of effect sizes by measuring all effects on a common scale, and centering makes the main effects more biologically interpretable when interactions are present [60, 61]. Prior to the beginning of data analysis, we excluded four captive hyenas from the analysis of anti-KLH natural antibodies because they were immunized with KLH one year prior to the collection of the serum samples used in this study; anti-KLH IgG and IgM levels in these individuals had already returned to pre-immunization levels [62], but, nevertheless, we excluded these animals because their anti-KLH antibodies no longer satisfied the criteria for natural antibodies. One wild hyena was excluded from the study prior to beginning data analysis because its serum contained a large lipid globule and could not be filtered in a manner consistent with the other serum samples. Additionally, we ran out of sera from two wild hyenas prior to performing the anti-ANA IgM assay so their values are not represented in that assay.

We also performed univariate non-parametric tests of the effects of captivity on immune function and the results were similar to the AICc model selection approach. Correlations among total IgG, total IgM, anti-ANA IgG, anti-ANA IgM, anti-KLH IgG, anti-KLH IgM, and BKC were assessed using Pearson product-moment correlation tests. Differences in variability between immune measures in captive and wild hyenas were assessed using Levene’s test for homogeneity of variance.

## Results

### Total IgG and IgM

**Fig 2:**
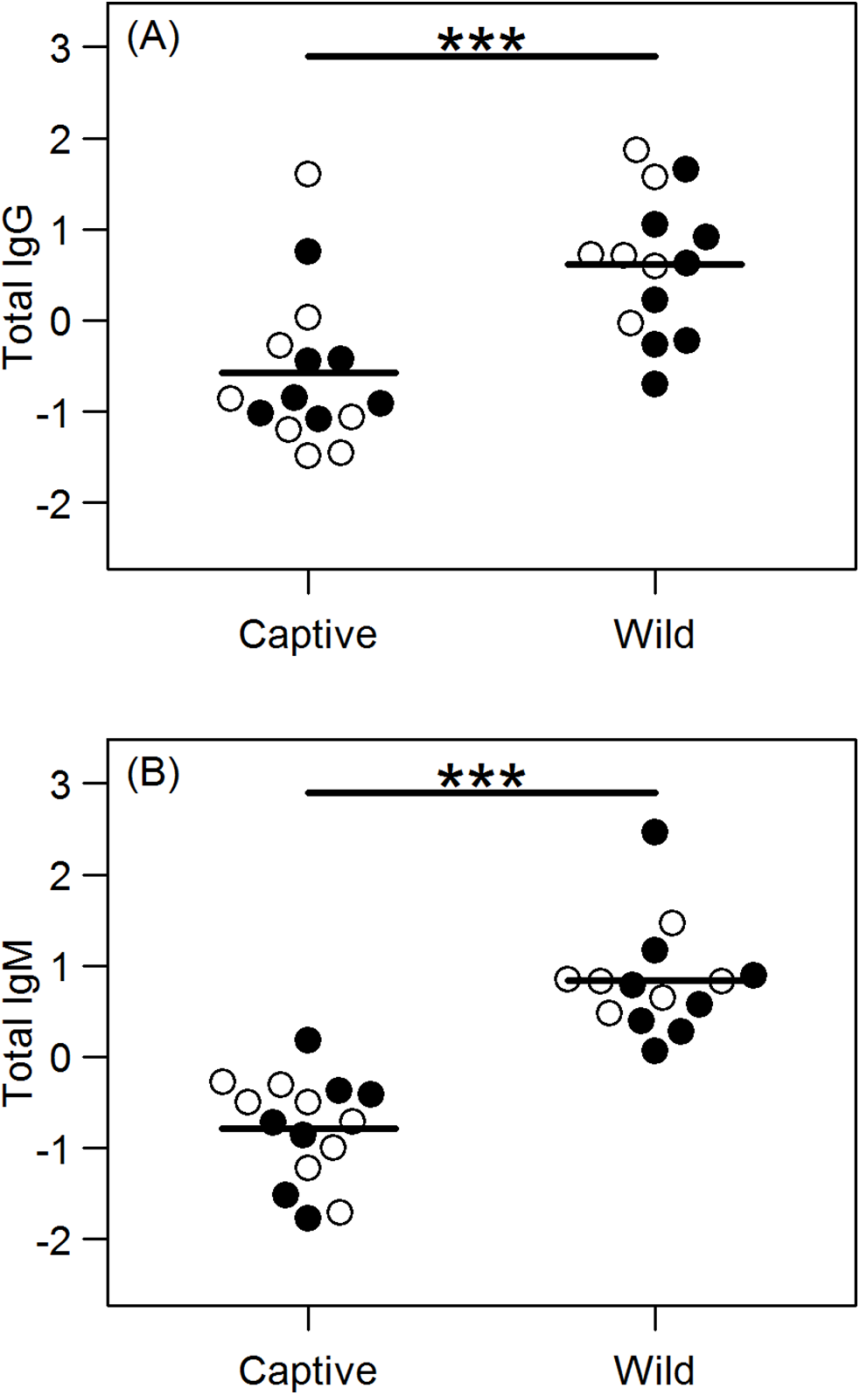
Relative concentrations of total IgG and IgM. Total IgG (A) and total IgM (B) are significantly higher in wild hyenas (n = 14) than in captive hyenas (n = 15). Females are indicated by open circles and males by filled circles. ***p < 0.001.

We assessed the relative concentrations of total IgG and IgM in 15 captive and 14 wild hyenas. For total IgG, only a single model had Δ AICc < 2; captivity status was the only input variable in this model, indicating the wild hyenas had significantly higher serum concentrations of total IgG than did captive hyenas (p < 0.001) (Fig 2A). Wild hyenas also had significantly more total IgM than captive hyenas (p < 0.001) (Fig 2B); here age was non-significant (p = 0.100), but was included in the top model set (Δ AICc < 2). (See S1 Table for full list of effect sizes, confidence intervals, and p-values for the analysis of total IgG and IgM).

### Natural IgG and IgM against KLH

**Fig 3:**
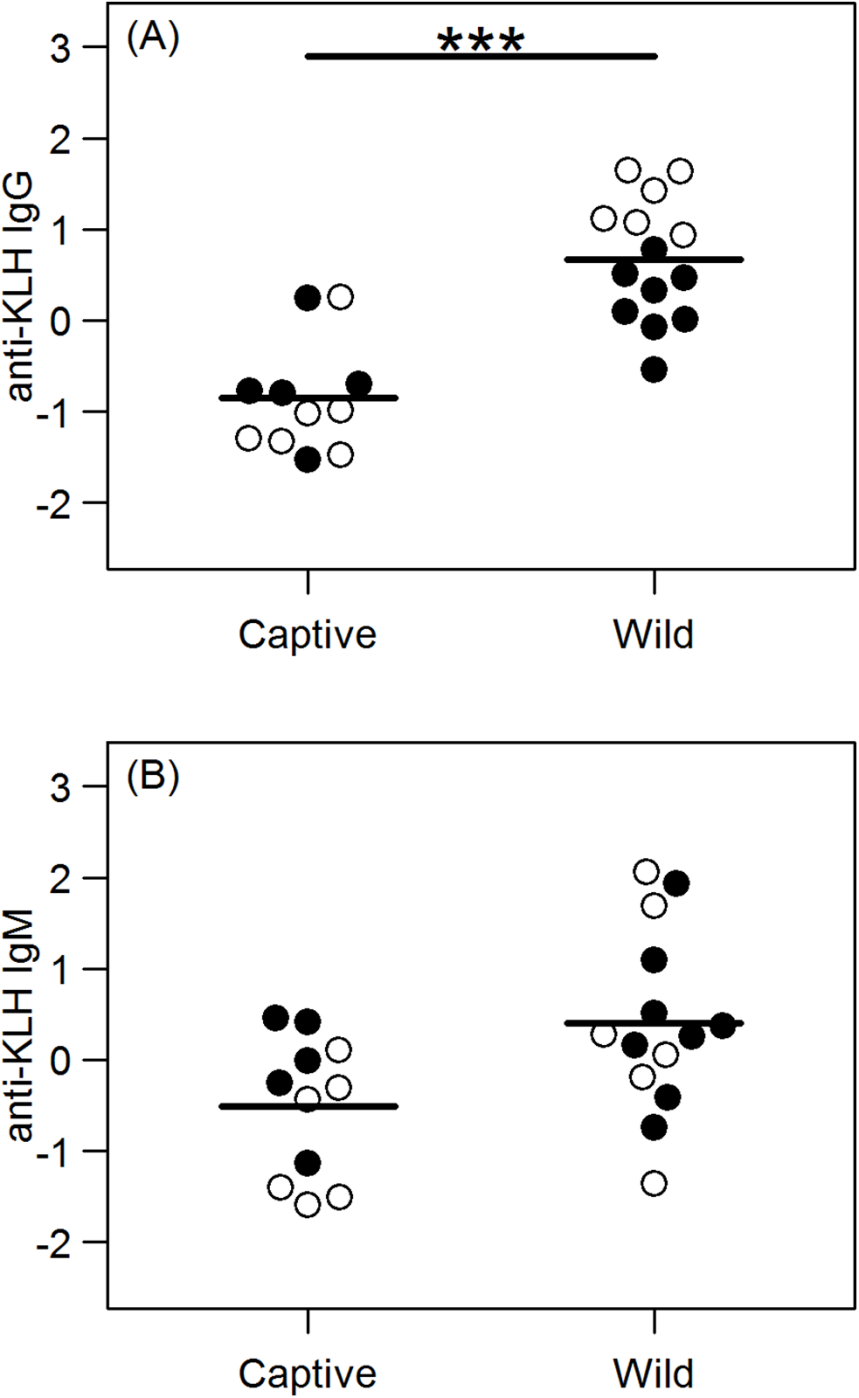
Relative concentrations of anti-KLH natural antibodies. Anti-KLH natural IgG (A) is significantly higher in wild hyenas (n = 14) than in captive hyenas (n = 11), but there is no difference in anti-KLH natural IgM (B) between wild (n = 14) captive (n = 11) hyenas. Females are indicated by open circles and males by filled circles. *** *p* < 0.001.

We measured natural antibodies to KLH in sera from 11 captive hyenas and 14 wild hyenas. As with total IgG, there was again only a single model with Δ AICc < 2, however, this time the model included captivity status, sex, and an interaction between captivity status and sex. Wild hyenas had significantly higher concentrations of anti-KLH nIgG than captive hyenas (p < 0.001) (Fig 3A). There was also a significant interaction between captivity status and sex (p = 0.003), with all wild female hyenas having higher concentrations of anti-KLH nIgG than wild male hyenas (Fig 3A). The weighted averages from the four anti-KLH nIgM models with Δ AICc < 2 suggest that there was no statistically significant difference (p = 0.116) between anti-KLH nIgM concentrations in wild hyenas than captive hyenas (Fig 3B). a marginally non-significant interaction between sex and age (p = 0.051), with anti-KLH nIgM decreasing with age in females. (See S2 Table for full list of effect sizes, confidence intervals, and p-values for the analysis of natural anti-KLH nIgG and nIgM)

### Autoreactive IgG and IgM

**Fig 4:**
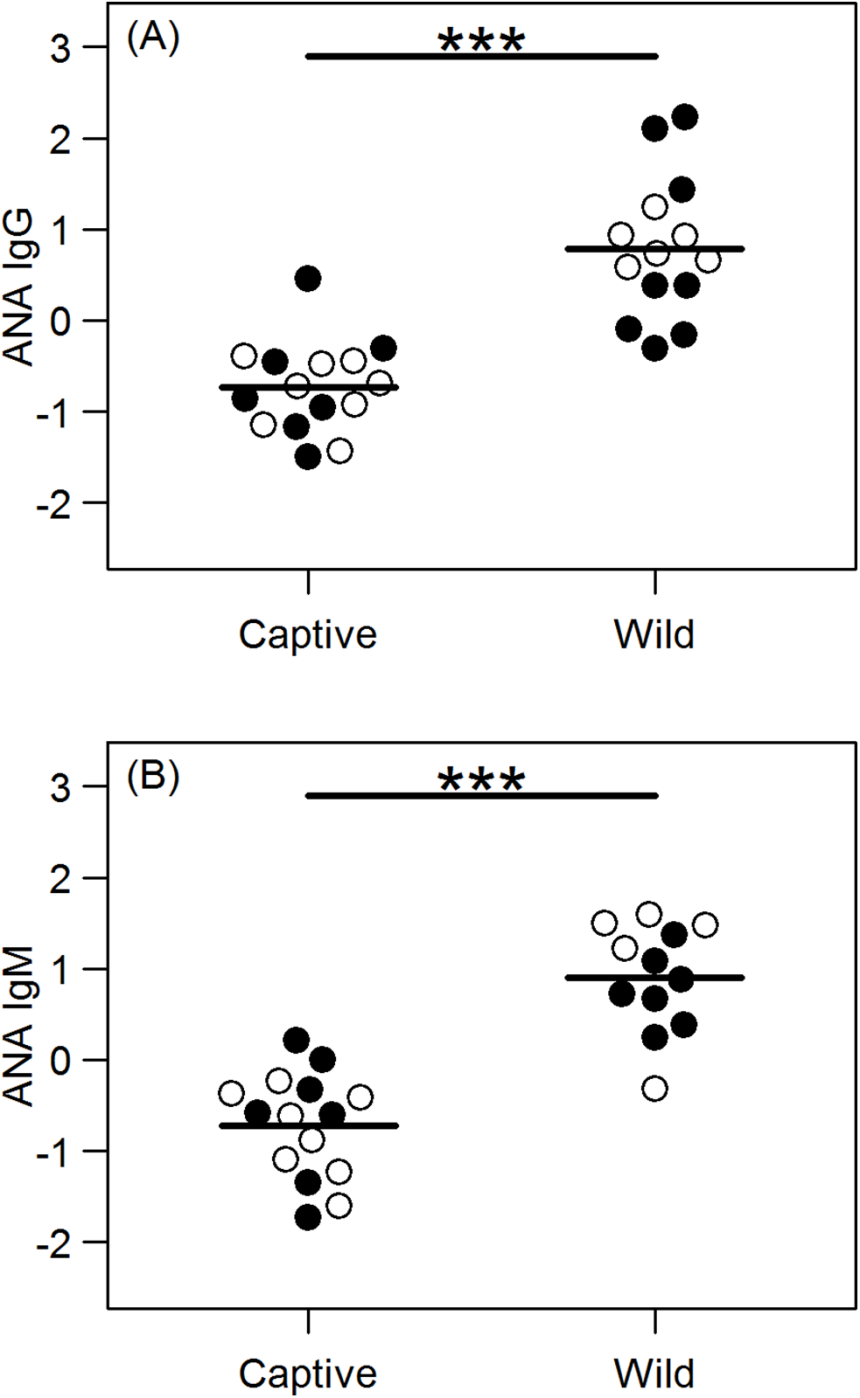
Relative concentrations of anti-nuclear antibodies. Anti-nuclear IgG (A) and anti-nuclear IgM (B) are significantly higher in wild hyenas (n = 14 for IgG; n = 12 for IgM) than in captive hyenas (n = 15 for IgG and IgM). Females are indicated by open circles and males by filled circles. *** *p* < 0.001.

We assessed the relative concentrations of aIgG in 15 captive and 14 wild hyenas, and aIgM in 15 captive and 12 wild hyenas. Wild hyenas had significantly higher levels of both aIgG (p < 0.001) and aIgM (p < 0.001) (Fig 4). aIgM decreased with age (p = 0.039), particularly in females. (See S3 Table for full list of effect sizes, confidence intervals, and p-values for the analysis of aIgG and aIgM)

### Bacterial killing capacity (BKC)

**Fig 5:**
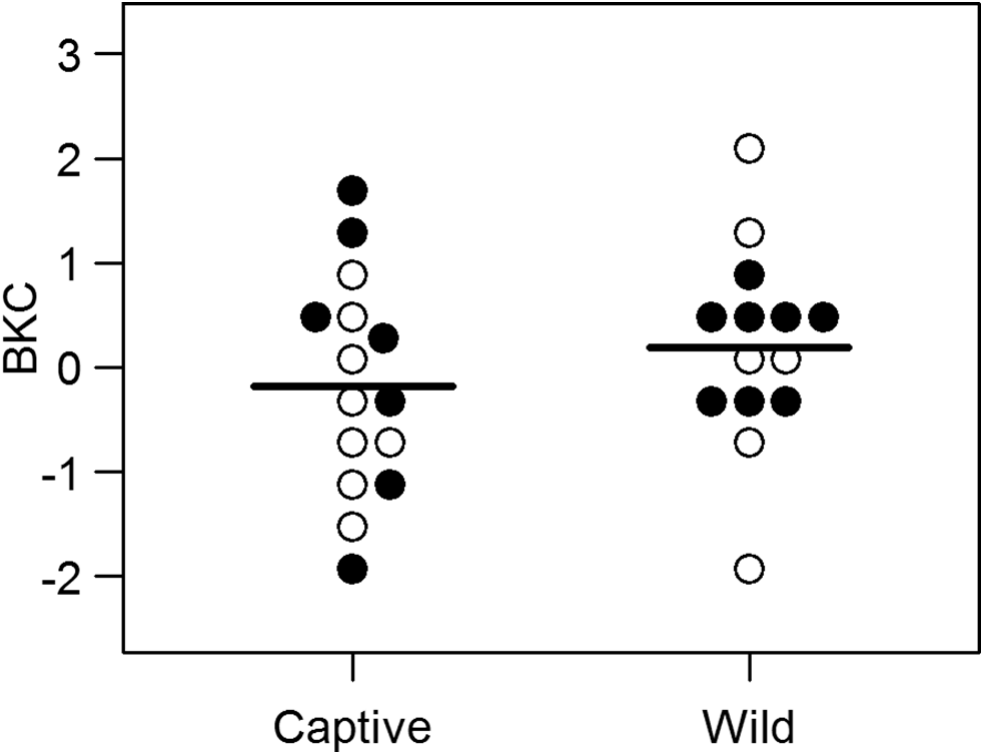
Bacterial killing capacity (BKC) of serum. BKC is similar in captive (n = 15) and wild (n = 14) hyenas. Females are indicated by open circles and males by filled circles.

We assessed BKC for 15 captive and 14 wild hyenas and found that none of the input variables or interactions significantly affected the BKC in the hyenas we tested. Captivity status and sex were included in the best fitting model set (Δ AICc < 2), but neither captivity status (p = 0.336) nor sex (p = 0.433) significantly affected BKC (Fig 5). The null model, which included only the intercept, had the lowest AICc value. (See S4 Table for full list of effect sizes, confidence intervals, and p-values for the analysis of BKC)

### Homogeneity of variance and correlations among immune measures

Levene’s tests revealed no difference with respect to homogeneity of variance between captive and wild hyenas for any immune measure (p > 0.45 in all cases), indicating that variance in immune measurements was homogenous across all samples. Additionally, see Table 1 for a full list of correlations among immune measures.

**Table 1:** Correlations among antibody concentrations and BKC.

Parameter 1	Parameter 2	r	d.f	P
	total IgM	0.344	27.000	*0*.*068*
	nAb IgG	0.421	23.000	**0.036**
total IgG	nAb IgM	-0.520	23.000	0.805
	ANA IgG	0.550	27.000	**0.002**
	ANA IgM	0.389	25.000	**0.045**
	BKC	-0.001	27.000	0.995
	nAb IgG	0.217	23.000	0.298
	nAb IgM	0.464	23.000	**0.019**
total IgM	ANA IgG	0.134	27.000	0.488
	ANA IgM	0.734	25.000	**0.001**
	BKC	0.113	27.000	0.558
	nAb IgM	0.131	23.000	0.533
nAb IgG	ANA IgG	0.332	23.000	*0*.*105*
	ANA IgM	0.614	21.000	**0.002**
	BKC	0.023	23.000	0.911
	ANA IgG	-0.008	23.000	0.971
nAb IgM	ANA IgM	0.488	21.000	**0.018**
	BKC	0.255	23.000	0.219
ANA IgG	ANA IgM	0.392	25.000	**0.043**
	BKC	0.075	27.000	0.698
ANA IgM	BKC	0.046	25.000	0.820

## Discussion

Here we show that wild hyenas inhabiting a pathogen-rich environment have higher levels of induced immune defenses than captive hyenas, whereas we observed no significant difference between wild and captive hyenas with respect to the constitutive immune defenses we tested. Our study is one of the first to use a non-rodent species to make comparisons of antibody concentrations using wild and wild-derived animals that are genetically similar. Importantly, our captive population was derived directly from the wild population, and has been maintained in captivity for less than four generations.

Previous research that compared immunoglobulin levels among human populations reported that total IgM concentrations were greater in populations where infectious disease prevalence was higher [63, 64], suggesting the environmental factors can influence total IgM concentrations. However, several studies comparing laboratory mice inhabiting antigen-free, germ-free, or specific pathogen-free environments detected no difference in total IgM concentrations in the three different environments, suggesting that in mice IgM is primarily a constitutive defense that is not affected by environment [65–67]. Similarly, Devalapalli, Lesher, et al. [15] found no difference in total IgM levels between laboratory mice and wild mice. However, Devalapalli, Lesher, et al. [15] reported significantly greater concentrations of total IgM in wild-caught rats than in laboratory rat strains, and these studies have recently been extended to demonstrate that biome enrichment are important for the development of the humoral immune system [12]. Here we found that total IgM concentrations are significantly greater in wild hyenas than in a captive population. Collectively, these studies indicate that total IgM concentrations in some species may be affected by their environment, and highlight the importance of using multiple model systems, including some natural models, when attempting to understand the role of the immune system in health and disease.

Here we observed significantly greater nIgG against KLH in wild than in captive hyenas. Previous research in mice showed that approximately 20% of the expanded nAb-producing B-1 cell population showed an activation-induced cytidine deaminase IgM to IgG isotype switch [68]. Repeated stimulation of B-1 cells could lead to a higher proportion of nIgG-producing B-1 cells compared to nIgM-producing B-1 cells, and competition for antigen could further augment production of nIgG and decrease production of nIgM. This might account for the lack of a significant difference in nIgM against KLH in the captive and wild hyenas, and suggests that over the long-term nIgM is the least inducible antibody type described in Fig 1. However, quantification of nAbs against targets other than KLH and a larger sample size would provide greater statistical power to detect differences in nIgM and would help to further clarify this issue.

Interestingly, wild females had higher concentrations of nIgG against KLH than did the wild males, but this difference was not apparent in captive hyenas. Previous work has shown that nAbs, particularly natural autoantibodies, are important for the establishment of self-tolerance [34, 69, 70]. Very little is known about self-tolerance and autoimmunity in wild animals, but in humans, autoimmune diseases are more common in women than men, and are often associated with hormonal changes associated with pregnancy and tolerance to fetal antigens [71].

Despite the positive correlation between autoantibody levels and some autoimmune diseases such as systemic lupus erythematosus, high levels of aIgG are associated with a reduced risk of Alzheimer’s disease, Parkinson’s disease, and multiple sclerosis [72]. In a well-studied wild population of Soay sheep (*Ovis aries*) females had higher levels of aIgG than did males, and aIgG-positive females were more likely to survive harsh winters, had higher offspring survival, but reduced fecundity than did autoantibody-negative females [51], again demonstrating a context-dependent positive role for aIgG. Interestingly, in the study of wild of the Soay sheep the concentration of serum ANAs was positively correlated with the concentration of antibodies that bind antigens derived from an important parasitic trematode [51]. No parasitic helminths have been detected in the captive hyena colony in nearly 30 years by either fecal flotation or during postmortem assessment of the GI tract, whereas every fecal sample ever tested from wild hyenas harbored at least one genus of parasitic helminth egg, and many samples contained thousands of eggs [23].

Here we show that both aIgG and aIgM levels are significantly higher in wild hyenas than in captive hyenas. Low-affinity aAbs, which may be derived from B-1 or marginal zone B cells, might be critically important for maintenance of physiological homeostasis in wild hyenas that are faced with a relentless assault of viral pathogens, because aAbs are critical for efficient clearance of virally-infected apoptotic and necrotic cells. Further research is needed to differentiate between high- and low-affinity ANAs in hyenas, and this could provide much needed insight into the relationship between autoimmunity and pregnancy in female mammals.

Surprisingly, nIgM and aIgM were negatively correlated with age in females, and there was no association between any measure of IgG and age. Several studies have found that nIgG and aIgG increase with age [48, 73, 74]. It is possible that isotype switching from IgM to IgG may account for the decreased nIgM and aIgM observed in older hyenas.

An alternative to the hypothesis that differences in pathogen exposure lead to the observed differences in induced immune function observed in wild and captive hyenas is that resource availability varies between the two populations and could drive the observed differences. The captive hyenas used in this study were fed a standard diet of 0.5–1 kg of carnivore chow and approximately 0.5 kg of meat and bone daily, which is adequate for maintaining stable body mass. Several days may pass between meals for wild hyenas, and their body masses are highly variable, depending on the size and timing of the last meal. If resources are the primary mediator of the immune defenses tested here, then we would expect to see more variable immune defense levels in wild hyenas. However, our results indicate that variance in immune defenses was similar among captive and wild hyenas. Nevertheless we would need serum samples collected at multiple time points from the same individuals to rule out this hypothesis altogether.

Currently there is a dearth of data in regard to allergy and autoimmunity in animals other than humans and rodents, but collaborative studies between wildlife biologists, immunologists, and managers of captive animals could greatly expand the range of comparative work in this area. Importantly, the groundwork for this type of comparative research is already in place in the form of zoos and wildlife research studies that routinely collect tissue samples from captive animals and keep accurate records of the medical history for each animal. The feasibility and impact of comparative immunology research should be augmented by the rapidly increasing availability of published genomes and decreasing costs of monoclonal antibody production, which should allow reagents, such as isotype subclass specific antibodies, to be developed in a cost-effective manner for non-traditional study species. The recent discovery of the important role of TLR10, which is expressed in human and hyenas but not in mice [75], in the innate immune response to influenza virus in humans [76] suggests that some infectious disease research might progress more rapidly by incorporating comparative studies of non-traditional species. However, in future studies, we do caution that the genetic distance between the captive and wild populations should be minimal, and meticulous monitoring and record keeping are needed to establish unambiguous differences between the captive and wild populations.

## Conclusions

This work shows that wild hyenas from the Masai Mara in Kenya had significantly higher serum antibody concentrations than hyenas born and raised in a captive facility. Importantly, the captive hyena population was derived directly from the wild population and has been in captivity for less than four generations. The striking differences observed here suggest that environmental and ecological factors are important modulators of the immune response, and that comparative analysis of captive and wild animals may help to uncover patterns that are not apparent in traditional laboratory studies of immune defenses.

## Supporting Information

S1 Dataset Spreadsheet containing the data used in this study. (CSV)

S1 Table Results of AICc based multimodel weighted-averages for total IgG and IgM. (DOCX)

S2 Table Results of AICc based multimodel weighted-averages for natural anti-KLH IgG and IgM. (DOCX)

S3 Table Results of AICc based multimodel weighted-averages for anti-ANA IgG and IgM. (DOCX)

S4 Table Results of AICc based multimodel weighted-averages for bacterial killing capacity (BKC). (DOCX)

## Abbreviations

nAb: natural antibody
ANA: anti-nuclear antibody
aAb: autoantibody
BKC: bacterial killing capacity
MIC: minimum inhibitory concentration, wild immunity
