# Supplementary material for: Markedly Elevated Antibody Responses in Wild versus Captive Spotted Hyenas Show that Environmental and Ecological Factors Are Important Modulators of Immunity
Source: PLoS One. 2015 Oct 7;10(10):e0137679. doi: 10.1371/journal.pone.0137679 (PMC4621877; doi:10.1371/journal.pone.0137679)
Supplement: S1 Table — (DOCX) [file pone.0137679.s002.docx]

| S1 Table. Results of AICc based multimodel weighted-averages for total IgG and IgM | | | | | | | |
| --- | --- | --- | --- | --- | --- | --- | --- |
| Response | Predictor | β | SE | Lower CI | Upper CI | p | Importance |
| Total IgG | Intercept | -0.579 | 0.208 | -1.006 | -0.151 | 0.010 | - |
|  | CS | 1.198 | 0.300 | 0.583 | 1.814 | < 0.001 | 1.000 |
| Total IgM | Intercept | -0.709 | 0.170 | -1.056 | -0.363 | < 0.001 | - |
|  | CS | 1.469 | 0.275 | 0.909 | 2.029 | < 0.001 | 1.000 |
|  | Age | -0.228 | 0.132 | -0.499 | 0.044 | 0.100 | 0.553 |

In cases where only a single model had Δ AICc < 2, the results from the single linear model are reported. CS = Captivity status.
