# Supplementary material for: Markedly Elevated Antibody Responses in Wild versus Captive Spotted Hyenas Show that Environmental and Ecological Factors Are Important Modulators of Immunity
Source: PLoS One. 2015 Oct 7;10(10):e0137679. doi: 10.1371/journal.pone.0137679 (PMC4621877; doi:10.1371/journal.pone.0137679)
Supplement: S2 Table — (DOCX) [file pone.0137679.s003.docx]

| S2 Table. Results of AICc based multimodel weighted-averages for natural anti-KLH IgG and IgM | | | | | | | |
| --- | --- | --- | --- | --- | --- | --- | --- |
| Response | Predictor | β | SE | Lower CI | Upper CI | p | Importance |
| anti-KLH IgG | Intercept | -0.974 | 0.204 | -1.398 | -0.550 | 0.000 | - |
|  | CS | 2.281 | 0.288 | 1.681 | 2.880 | < 0.001 | 1.000 |
|  | Sex | 0.263 | 0.302 | -0.366 | 0.891 | 0.394 | 1.000 |
|  | Sex * CS | -1.374 | 0.405 | -2.217 | -0.532 | 0.003 | 1.000 |
| anti-KLH IgM | Intercept | -0.135 | 0.361 | -0.863 | 0.593 | 0.716 | - |
|  | Age | -0.591 | 0.350 | -1.295 | 0.114 | 0.100 | 0.767 |
|  | CS | 0.737 | 0.449 | -0.183 | 1.656 | 0.116 | 0.427 |
|  | Sex | -0.089 | 0.375 | -0.870 | 0.691 | 0.822 | 0.193 |
|  | Sex * Age | 0.823 | 0.397 | -0.002 | 1.648 | 0.051 | 0.193 |

In cases where only a single model had Δ AICc < 2, the results from the single linear model are reported. CS = Captivity status.
