# Supplementary material for: Markedly Elevated Antibody Responses in Wild versus Captive Spotted Hyenas Show that Environmental and Ecological Factors Are Important Modulators of Immunity
Source: PLoS One. 2015 Oct 7;10(10):e0137679. doi: 10.1371/journal.pone.0137679 (PMC4621877; doi:10.1371/journal.pone.0137679)
Supplement: S3 Table — (DOCX) [file pone.0137679.s004.docx]

| S3 Table. Results of AICc based multimodel weighted-averages for anti-ANA IgG and IgM | | | | | | | |
| --- | --- | --- | --- | --- | --- | --- | --- |
| Response | Predictor | β | SE | Lower CI | Upper CI | p | Importance |
| ANA IgG | Intercept | -0.754 | 0.176 | -1.114 | -0.393 | 0.000 | - |
|  | CS | 1.561 | 0.266 | 1.016 | 2.106 | < 0.001 | 1.000 |
|  | Age | 0.124 | 0.154 | -0.191 | 0.440 | 0.441 | 0.270 |
| ANA IgM | Intercept | -0.452 | 0.195 | -0.850 | -0.055 | 0.026 | NA |
|  | CS | 1.241 | 0.240 | 0.745 | 1.738 | < 0.001 | 1.000 |
|  | Age | -0.517 | 0.244 | -1.008 | -0.026 | 0.039 | 1.000 |
|  | Sex | -0.227 | 0.195 | -0.632 | 0.178 | 0.272 | 0.550 |
|  | Sex * Age | 0.491 | 0.210 | 0.054 | 0.927 | 0.027 | 0.550 |

In cases where only a single model had Δ AICc < 2, the results from the single linear model are reported. CS = Captivity status.
