# Supplementary material for: Markedly Elevated Antibody Responses in Wild versus Captive Spotted Hyenas Show that Environmental and Ecological Factors Are Important Modulators of Immunity
Source: PLoS One. 2015 Oct 7;10(10):e0137679. doi: 10.1371/journal.pone.0137679 (PMC4621877; doi:10.1371/journal.pone.0137679)
Supplement: S4 Table — (DOCX) [file pone.0137679.s005.docx]

| S4 Table. Results of AICc based multimodel weighted-averages for bacterial killing capacity (BKC) | | | | | | | |
| --- | --- | --- | --- | --- | --- | --- | --- |
| Response | Predictor | β | SE | Lower CI | Upper CI | p | Importance |
| BKC | Intercept | -0.081 | 0.241 | -0.573 | 0.411 | 0.748 | - |
|  | CS | 0.374 | 0.372 | -0.388 | 1.136 | 0.336 | 0.257 |
|  | Sex | 0.307 | 0.374 | -0.460 | 1.074 | 0.433 | 0.216 |

CS = Captivity status.
